# A sequential explanatory study of the employment experiences of population-based breast, colorectal, and prostate cancer survivors

**DOI:** 10.1007/s10552-021-01467-5

**Published:** 2021-06-27

**Authors:** Shoshana Adler Jaffe, Dolores D. Guest, Andrew L. Sussman, Charles L. Wiggins, Jessica Anderson, Jean A. McDougall

**Affiliations:** 1 University of New Mexico Comprehensive Cancer Center, Albuquerque, NM; 2 Department of Internal Medicine, University of New Mexico, Albuquerque, NM; 3 Department of Community and Family Medicine, University of New Mexico, Albuquerque, NM; 4 New Mexico Tumor Registry, Albuquerque, NM

**Keywords:** Cancer, Mixed-Methods, Survivors, Caregivers, Employment

## Abstract

**Purpose:**

Cancer treatment often leads to work disruptions including loss of income, resulting in long-term financial instability for cancer survivors and their informal caregivers.

**Methods:**

In this sequential explanatory study, we conducted a cross-sectional survey of employment experiences among ethnically diverse, working-age individuals diagnosed with breast, colorectal, or prostate cancer. Following the survey, we conducted semi-structured interviews with cancer survivors and informal caregivers to explore changes in employment status and coping techniques to manage these changes.

**Results:**

Among employed survivors (n=333), cancer caused numerous work disruptions including issues with physical tasks (53.8%), mental tasks (46.5%) and productivity (76.0%) in the workplace. Prostate cancer survivors reported fewer work disruptions than female breast and male and female colorectal cancer survivors. Paid time off and flexible work schedules were work accommodations reported by 52.6% and 36.3% of survivors, respectively. In an adjusted regression analysis, household income was positively associated with having received a work accommodation. From the qualitative component of the study (survivors n=17; caregivers n=11), three key themes emerged: work disruptions, work accommodations, and coping mechanisms to address the disruptions. Survivors and caregivers shared concerns about lack of support at work and resources to navigate issues caused by changes in employment.

**Conclusions:**

This study characterized employment changes among a diverse group of cancer survivors. Work accommodations were identified as a specific unmet need, particularly among low-income cancer survivors. Addressing changes in employment among specific groups of cancer survivors and caregivers is critical to mitigate potential long-term consequences of cancer.

## 1. Background:

A cancer diagnosis often has detrimental effects on the employment for patients and their caregivers. Forty-six percent of all cancer diagnoses occur in working-age adults between the ages of 20 and 64.[1] The diagnosis and treatment of cancer often requires that patients take time away from work. Results from one study, for example, indicated that the value of patients’ time associated with travel to and from care, waiting for appointments, and time spent receiving care was $4,592, on average, in the first year after a diagnosis of colorectal cancer.[2] In addition, the lasting effects of treatment, including fatigue, neuropathy, and neuropsychological impairment, can have long-term impacts on ability to work and productivity.[3]

This combination of increased medical expenditures, time costs, and decreased income and productivity leads to substantial financial hardship, including accumulating debt, filing for bankruptcy, making financial sacrifices, and being unable to cover the cost of medical bills.[4] Prior studies indicate an inverse association between age and financial hardship[5–7], suggesting that working-age cancer survivors may be particularly vulnerable to the financial shock of a cancer diagnosis.

Cancer caregivers also experience substantial work disruptions and negative financial consequences. Informal/family caregivers include family members, significant others, and friends that manage care that is typically uncompensated, delivered at home, involves significant amounts of time and energy, and requires the performance of tasks that may be physically, emotionally, socially, or financially demanding.[8] In a recent review, mean out-of-pocket costs associated with caregiving, including travel expenses and medication or care supplies, were estimated to be $447 per month, with another $206 per month in lost productivity at work and $4,809 per month in time costs.[9] In a population-based survey of employed partners of breast cancer patients, 32% of partners reported decreasing their work hours, resulting in worsening financial status attributable to their partner’s cancer diagnosis.[10]

Studies examining the relationship between cancer treatment and employment have found that workplace accommodations, flexible work schedules and paid time off are associated with job retention. [11–13] Yet, systematic differences in the type of accommodation needed exist by job sector. There is also evidence that workplace accommodations have a disproportionately positive effect among women who perform mental, rather than physical, tasks as part of their job.[14] The diversity of occupations and systemic disparities in benefits tied to employment, with women and minorities overrepresented in jobs that pay <$15 per hour [15], highlights the importance of characterizing employment outcomes among understudied populations.[11] Moreover, research targeting medically underserved and underrepresented patient populations and the unmet needs of caregivers in these populations is an important research priority.[8] In addition, further research is needed in the Hispanic community to inform efforts to develop targeted interventions.

To address these gaps in our understanding of employment disruptions and accommodations among diverse cancer survivors and caregivers, we used a sequential explanatory study design[16] to characterize the employment experiences of a population-based sample of cancer survivors and informal caregivers in New Mexico.

## 2. Methods:

Using a sequential explanatory study design, we collected and analyzed quantitative and then qualitative data in two consecutive phases within one study, the Comprehensive History of Individuals’ Cancer Experiences (CHOICE) Project. [17]

### 2.1 Quantitative data collection and analysis:

The first phase of the CHOICE Project consisted of a cross-sectional survey of cancer survivors identified from the New Mexico Tumor Registry (NMTR). Individuals diagnosed between 2008 and 2016 with stage I-III female breast, colorectal (CRC), or prostate cancer, between the ages of 21–64 years, were sampled on the basis of their insurance status at diagnosis. All Medicaid and uninsured patients with an income <200% of the Federal Poverty Level were included, and a random 1:1 sample of privately insured patients matched by year of diagnosis.

The survey was provided to eligible individuals in English and Spanish, using identical paper, web-based, and computer assisted telephone interview (CATI) formats, based on participant preference. Employment status and subsequent changes were assessed through self-reported responses to validated questions from the Medical Expenditure Panel Survey Experiences with Cancer Supplement.[18] Consistent with previous literature [12, 13], we categorized changes in employment occurring anytime from diagnosis to the time of the survey into three broad categories: 1) work disruptions, including change from a full-time to part-time position, cancer interference with physical tasks at work, cancer interference with mental tasks at work, and cancer interference with productivity at work); 2) work accommodations, including change from a set schedule to a flexible schedule, taking extended paid time off and, 3) remaining at a job to keep health insurance. These categories were not mutually exclusive. The survey asked participants to detail their employment history since the time of their cancer diagnosis. In addition, the survey included questions about whether a cancer survivor had an informal caregiver, and if so, if that caregiver made work changes and the duration of any caregiver work changes.

Information on covariates including current comorbid conditions, highest level of education obtained, insurance status during cancer treatment, current income, debt in the year prior to diagnosis (excluding car loans and mortgages), current marital status and ethnicity were obtained through self-report. Age at diagnosis, year of diagnosis, cancer site, sex, race, and residential zip code were ascertained from NMTR records. Rural-Urban Commuting Area (RUCA) codes were used to classify those who resided in a rural area based on current mailing address.[19] Due to small numbers, those with no insurance were combined with Medicaid insured patients.

We used univariate logistic regression to estimate odds ratios (OR) and 95% confidence intervals (CI) for the association between sociodemographic characteristics and work disruptions and work accommodations. Multivariable logistic regressions were used to estimate the OR and 95% CI for the association between work disruptions as well as work accommodations. Using backward stepwise regression and retaining variables that remained significant at the p<0.20 level, we built a multivariable logistic regression model for each exposure of interest. Due to clinical significance, age was included in both of the final models. All data analysis was conducted in Stata version 15.1.[20]

### 2.2 Qualitative data collection and analysis:

**Fig 1:**
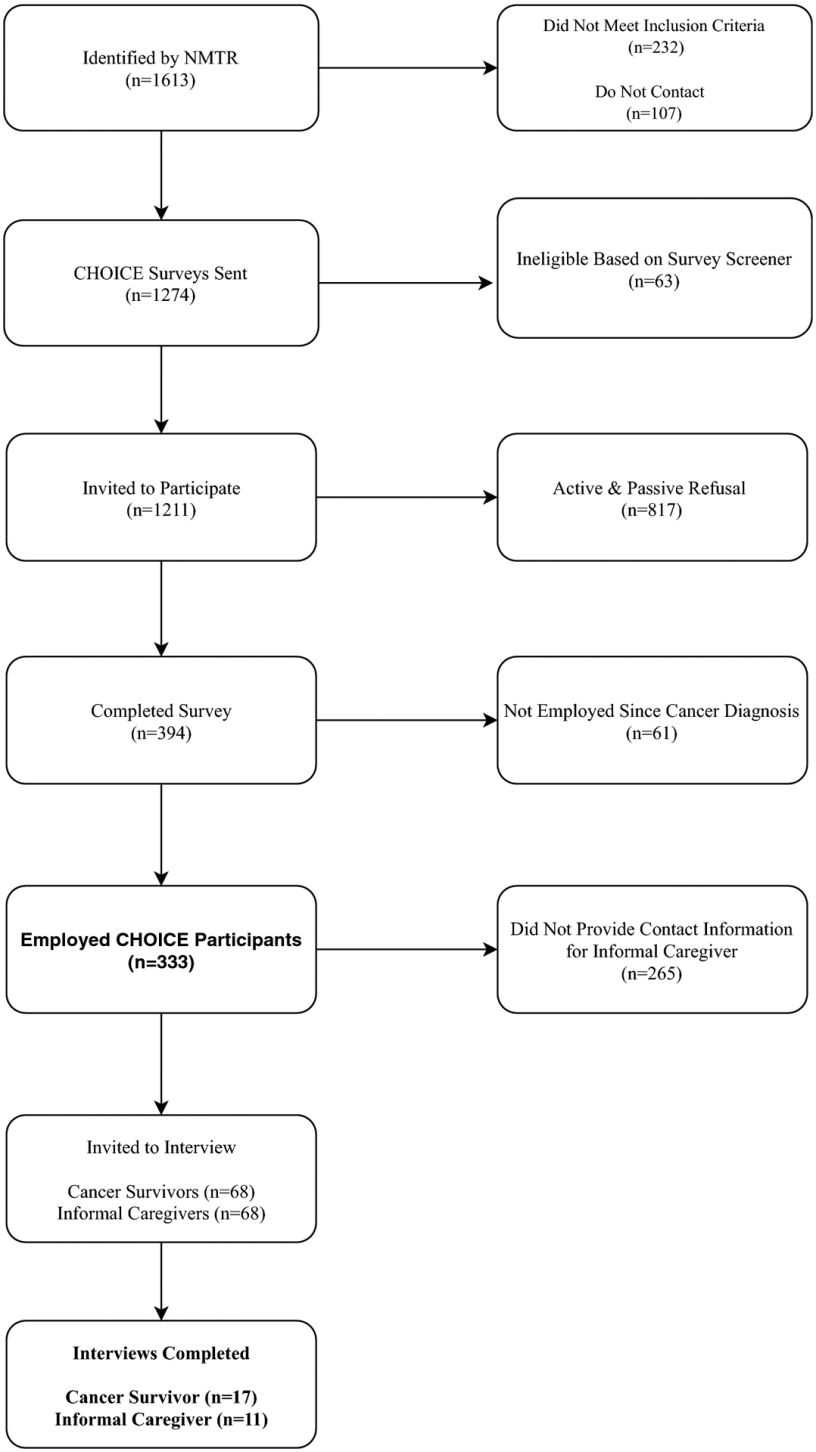
Consort Diagram

Following the quantitative survey data collection and analysis, we sampled cancer survivors who provided contact information for their named informal caregiver to participate in separate semi-structured phone interviews (Figure 1). We did not require that both the cancer survivor and caregiver complete an interview to be included in the final analysis.

Using two separate interview guides, one for cancer survivors and one for informal caregivers, we investigated individual experiences with work disruptions, accommodations, and remaining at a job to keep insurance. In addition, we sought information about coping behaviors or strategies that cancer survivors and caregivers used to address the financial burden associated with changes in employment and the impact of these changes on their quality of life.

We determined that we reached information saturation when no new themes were identified during data analysis.[21] Interviews were audio recorded and recordings were transcribed and read independently by all members of the research team. Using the methodology detailed in Maguire and Delahunt’s thematic analysis guide [22], research team members reached consensus on a coding template. Three research team members completed the process of coding the transcripts, meeting weekly to ensure consistency of application and resolving conflicts. The coding team generated queries and summaries for review by the full research team to identify final thematic interpretations.

## Results:

### 3.1 Quantitative:

Out of 1,211 potentially eligible participants, 394 people completed the quantitative CHOICE survey (response rate 33%). Survey respondents were more likely to be female and non-Hispanic white and have a diagnosis of stage 1 breast cancer than non-respondents. Of those who took the survey, 333 (84.5%) individuals were employed (Figure 1). Among employed cancer survivors, the median age at diagnosis was 52 years (range: 27–62). The average time since diagnosis was 6 years. About 40% identified as Hispanic and nearly a quarter of participants had Medicaid or were uninsured (n=80). About 38% of participants reported having no comorbidities. More than half (54.6%) of those surveyed reported a current annual household income of less than $59,000. Participants who resided in a rural area made up 34% of the employed study population. Additional demographic characteristics stratified by cancer site and sex are presented in Table 1.

Overall, 31.8% of employed cancer survivors surveyed reported changing from a full time to part time position (Table 2). Cancer interfered with physical tasks (53.8%), mental tasks (46.5%) and productivity (76.0%) for a substantial number of employed cancer survivors. However, a distinct pattern in work disruptions reported by cancer type emerged, with prostate cancer survivors consistently reporting fewer work disruptions than female breast and male and female colorectal cancer survivors. Conversely, fewer prostate cancer survivors (13.7%) reported changing from a set to a flexible schedule than female breast (42.6%), male colorectal (45.2%), and female colorectal (40.6%) cancer survivors. Extended paid time off was also less common among male colorectal (38.7%) and prostate (39.7%) cancer survivors than among female breast (58.9%) and female colorectal (56.3%) cancer survivors.

Health insurance, which is frequently linked to employment, was cited as a reason to maintain a job among 42.9% of survivors. However, staying at a job due to concerns about losing health insurance was less common among male colorectal survivors (32.3%) as compared to female colorectal cancer survivors (56.3%). About the same proportion of prostate (43.8%) and breast cancer survivors (42.1%) reported staying at a job to retain health insurance coverage.

Of the 333 employed individuals, 259 (77.8%) indicated that they had an informal caregiver (Table 2). Work changes for the informal caregiver were reported by 30.3% of cancer survivors and 20.4% reported that their informal caregiver made a work change for at least 2 months. Overall, 13.8% of caregivers stayed at their job due to concerns about losing health insurance coverage for their family.

In the model adjusted for the covariates presented in Table 3, prostate cancer survivors were less likely to report a work disruption (OR 0.39, 95% CI 0.15, 0.98) than breast cancer survivors. Participants with an annual household income of $30,000-$69,000 were three times (OR 3.06, 95% CI 0.91,10.25) as likely to report a work disruption than survivors with an income of less than $30,000. Survivors with two or more comorbidities were more than three times (OR 3.59, 95% CI 1.27,10.1) as likely to report receiving a work accommodation than individuals with no comorbidities. Individuals with an income between $30,000-$69,000 were more than 5 times (OR 5.29, 95% CI 1.87,14.97) as likely to report a work accommodation and those with an income of more than $70,000 were 6 times (OR 6.07, 95% CI 2.14,17.20) as likely to indicate they experienced a work accommodation than those who made less than $30,000 annually.

### 3.2 Qualitative:

We reached saturation after interviewing a total of 28 participants (17 cancer survivors and 11 informal caregivers) in the qualitative phase of the study. The majority of the cancer survivors interviewed were female (88%), while the majority of informal caregivers interviewed were male (55%). About 60% of our cancer survivor interviewees had a current annual household income of less than $50,000. Among the informal caregivers we interviewed, all but one individual reported being the spouse or partner of a cancer survivor. Additional demographic data on the qualitative participants is presented in Table 4.

Semi-structured interviews yielded data that could be organized into three key categories: employment disruptions, employment accommodations, and coping techniques including staying at a job to maintain access to insurance and federal assistance programs for employed individuals. A summary of the themes that emerged, examples of the themes and the type of participant who reported such experiences is presented in Table 5.

#### 3.2.1 Employment Disruptions

Cancer survivors described two common types of workplace disruptions: inability to perform expected tasks and inability to maintain the same work schedule they had prior to diagnosis. For some participants, these disruptions eventually led to employment termination. For example, one cancer survivor stated that she could no longer work after her diagnosis because she was unable to perform the physical tasks required of her job, *“I couldn’t work cause I am a waitress and you can’t lift anything over 10 pounds [after surgery]…So I just could not work.”*

Many survivors expressed frustration regarding completing mental tasks at work and one survivor said, “*I go to work every day and they expect me to be normal and I’m not normal. They don’t get it and I don’t know how to explain it to them…no it’s the chemo brain…oh I forgot…. don’t get mad at me cause I forgot*.”

Work disruptions were also described as long-lasting issues. One survivor highlighted this point when she lamented the duration of her physical and mental limitations, saying: *“It’s been almost 5 years, so that was the other thing too, even though my job is not physical I still get tired.”*

Survivors also described disruptions to their regular work schedule because they were unable to work as many hours as they used to. This reduction in hours had a direct impact on their ability to afford necessities, as illustrated by this survivor’s experience, *“So I was a 5-day-a-week worker at the restaurant…I worked a lot of doubles. Then, I had to go to 2 days a week. I couldn’t make the mortgage payments. I could hardly pay any of my bills on 2 days a week. I have 3 kids.”*

Work disruptions were also a concern for informal caregivers. Caregivers reported taking time off of work to care for their loved one and facing serious repercussions for doing so. For example, one informal caregiver said, *“…if you’re not at work after 30 days then you don’t have no insurance. Without that insurance, she couldn’t get her treatments.”* This balancing act of taking time off while maintaining health insurance benefits was a recurrent issue among caregivers.

#### 3.2.2 Employment Accommodations

Both survivors and informal caregivers described multiple different types of accommodations offered to them by their place of employment. Many indicated that they felt “lucky” or “blessed” to have been provided such accommodations. Paid time off was one type of accommodation that survivors described as being unparalleled in helping them handle their cancer diagnosis and treatment, *“I did have to take off of work, but I had a lot of sick leave available to me through my job, so I didn’t have to go without pay.”*

Caregivers were also vocal about the importance of the accommodations their workplace provided them. One caregiver said, *“Any time I could, I would take time off work just to be with her. I had a somewhat flexible schedule, so I was able to do that…but towards the end it was causing a little bit of conflict with my employer.”* Another caregiver described his situation, saying,

“*She [survivor] wasn’t working so I had to try and get more hours at work and to offset that…but not too many hours that I wasn’t there to be there for her and also be able to take her to the doctor. So, it was a big balancing act of just trying to get more hours and get time off and use up any PTO [paid time off] I had and any vacation time.”*

The stress that caregivers felt as they tried to balance working and caring for their loved one was a prevalent, underlying theme.

#### 3.2.3. Remaining at a job to keep insurance

Another survivor highlighted the short-term disability policy that her employer offered to ensure she still had an income, albeit a reduced income, during the period of time she was unable to work. She said, *“Once I got the diagnosis and learned what the treatment plan was going to be, it was gonna be near impossible to keep up with a full-time job or any job really. So, I ended up going on short-term disability and that lasted a few months that was at reduced pay.”*

One survivor highlighted the health insurance policy that her employer provided, saying: *“I was lucky enough that my employer was very kind and paid for my health insurance premiums and continued to do so until I figured out if I was going to go back to work or not.”*

#### 3.2.4. Coping Behaviors and Strategies

Survivors and caregivers turned to federal assistance programs such as Medicaid and the Supplemental Nutrition Assistance Program (SNAP) to help them cope with the financial issues they were experiencing as a result of an employment disruption and lack of workplace accommodations. However, the interplay between being employed and applying for such assistance proved to be problematic for many.

One survivor said, *“We worked less because we were able to get on a Medicaid program.”* Another described the dilemma she and her husband were faced with, “*We were on the food stamp program for a little while…until he went back to work and started making a little more money then of course they took us off.”* In both of these examples, cancer patients had to balance the amount they worked to ensure they did not lose the critical benefits they were receiving.

However, in some cases, the benefits provided were still not sufficient and one couple was faced with a drastic choice as described here by the cancer survivor herself, *“So what we decided to do, which totally sucked, but we decided to get divorced on paper. So that I could qualify for Medicaid because I had myself and a dependent [a child].”*

Survivors and caregivers also reported turning to other resources to cope with the financial challenges of being unable to maintain a regular work schedule. One specific technique used by survivors were crowd-funding platforms. These funds were reportedly used to pay for non-medical expenses such as rent, food, and gas as explained by one survivor, “*I did a go-fund-me. It helped me pay my mortgage, pay for gas to go to treatment, pay my bills…[pay] to eat…”*

Family and friends were most frequently noted as the primary contributors to the funds, but some cancer survivors stated that members of their community who they did not know also donated. One survivor, when asked to provide advice to current cancer patients, described the benefits of crowdfunding saying: *“I would do a go-fund-me…people we didn’t even know donated…and if you have faith in g-d…it does wonders.”*

## 3. Discussion:

In this study of population-based breast, colorectal, and prostate cancer survivors and caregivers, employment disruptions were experienced by 73% of participants and 62% reported an employment accommodation. In addition, 43% of cancer survivors reported that they remained at a job to keep health insurance. Our population differs from previously studied populations in several important ways. More than 60% of our population reported at least one comorbidity. Individuals with chronic health conditions who are diagnosed with cancer may experience more barriers in returning to work than those without chronic health conditions.[23] In addition, those with lower household incomes at diagnosis have been found to have more issues successfully returning to work.[24] About a third of our population had a household income of $30,000 or less, 24% had Medicaid or no insurance, 40% were Hispanic and 34% lived in rural areas. Evidence suggests that our population may be at particularly high risk of experiencing difficulties returning to or maintaining employment after a cancer diagnosis.[24]

Work disruptions were common in our study, with 76% of survey participants reporting that cancer interfered with productivity at work. In another study of breast, colorectal, lung, prostate, and melanoma patients, only 32% reported a decrease in productivity.[25] However, that study surveyed an insured, primarily non-Hispanic white population, suggesting potential disparities in the impact of cancer on employment productivity. Difficulty performing both physical and mental tasks were highlighted in qualitative interviews. The long-term effects of cancer and its treatment often came as a surprise to cancer survivors and caused considerable frustration. Similarly, caregivers struggled to balance work commitments and taking time off to accompany patients to appointments and provide care at home. We found that 20% of caregivers made a change in their work for more than 2 months which is comparable to other recently published findings.[26]

Nearly half of our study population reported being able to take paid time off from work. Annual household income was positively associated with the likelihood of having received a work accommodation. This highlights an important socioeconomic disparity in employment outcomes which is further supported by our qualitative data. Many lower-income participants held positions that did not offer paid time off. The United States does not have universal policies surrounding paid time off or sick leave. Some groups face serious disparities when they need to take time off of work for medical reasons and policies that mandate paid time off are inadequate or non-existent in many organizations. The lack of job protection policies can lead to job loss which is often devastating both financially and emotionally after a cancer diagnosis. A lack of resources to navigate working with limitations caused by cancer is another structural issue that has been highlighted in previous literature about the multi-level interactions associated with working while undergoing cancer treatment.[27] A recent study of working-age breast cancer patients found that 55.1% of women reported taking paid time off.[28] This is very similar to the proportion of breast cancer patients in our study (59%) who reported taking extended paid time off. While we did not collect specific details such as type, sector, or duration of an individual’s occupation, it has been suggested that insurance status is an acceptable proxy for an individual’s socioeconomic status. [29, 30] In general, individuals with Medicaid or no insurance work at jobs that do not offer benefits such as insurance, sick leave, or other legal protections such as access to the benefits of the Family and Medical Leave Act.[13] In addition, previous studies have documented the importance of having a flexible workplace setting to facilitate a successful return to work after cancer treatment to prevent negative changes in employment status or work capabilities.[31, 32]

The struggle individuals faced in trying to access federal assistance programs highlights a gap in resources. Cancer survivors and informal caregivers reported being disincentivized to work because they would lose access to their benefits. However, this may be detrimental because work provides a sense of normalcy and purpose for many people during a cancer diagnosis.[33] A cancer diagnosis is inherently a stressful time and taking time off of work or having to quit a job may lead to additional stress. Policy changes to account for unexpected medical diagnoses are urgently needed to address this issue.

Our results also indicate that employment experiences differed by cancer site. Prostate cancer survivors in our study reported fewer work disruptions than breast and colorectal cancer survivors. This may be attributable to the treatment regimens for prostate cancer.[34] However, it is important to note that in a previous study, a diagnosis of prostate cancer was still found to cause significant detrimental employment outcomes.[35] This demonstrates that while the frequency and magnitude of employment changes differs by cancer site, the impact must be addressed in a cancer-site specific manner. Cancer patients who receive chemotherapy are also more likely to report work disruptions than those who do not receive chemotherapy.[12, 24, 36] None of the prostate cancer survivors in our study received chemotherapy. In addition, the prostate cancer cases in this study were of higher socioeconomic position (92% of prostate cases had private insurance and 70% had an annual household income greater than $60,000) compared to those with breast or colorectal cancer. Treatment modality, cancer site and sex should be considered when addressing employment disruptions.

Finally, our qualitative findings about survivor perspectives of employment changes align with other recent work in this area.[33, 37] The disruptions described clearly had a negative impact on participants’ quality of life. The accommodations described were cited as key reasons participants were able to persevere through the challenges of cancer treatment and recovery. Concerns about being able to perform physical and mental tasks required of their job were a common issue. In addition, a lack of perceived understanding and recognition on the employers’ part was frequently cited by cancer survivors. A recent study suggested increasing awareness of accommodations that cancer survivors need through legislative action and employer education programs [37] and our findings support the necessity of both of these steps. The caregiver perspectives we captured clearly demonstrated the lack of support caregivers feel they have access to. These issues have been previously quantified [38], but our study furthers these findings by providing more detailed, qualitative examples of the problems employed caregivers often face. The issues noted by caregivers highlights an unmet need in cancer care. Supporting those who support cancer patients is an essential part of a holistic approach to treating cancer. The same steps necessary to obtain appropriate workplace accommodations for survivors should be undertaken to ensure that caregivers are offered workplace flexibility and support during their loved one’s cancer treatment and transition to survivorship.

### 4.1 Study limitations:

It is important to consider the context in which this study was conducted. New Mexico is unique both demographically and geographically. Specifically, New Mexico is a majority-minority state with nearly 50% of the population being Hispanic and about 10% identifying as Native American.[39] The census classifies 12 of the 33 counties in New Mexico as mostly or completely rural and this accounts for about a quarter of the population of the state. This proportion of rural inhabitants is greater than the national average and even though the remaining counties are classified as urban, they are still relatively sparsely populated, with the largest county having a population density of only 570 people per square mile.[39] Thus, while our findings may not be generalizable to all cancer patients, our results capture important data about the cancer experiences of those residing in New Mexico and this data may help us better formulate interventions to assist those faced with the challenges we characterized. We also asked individuals to detail their employment history from the time they were diagnosed through the time they took the survey. This may have resulted in recall bias if participants misattributed employment changes that occurred outside the specified time frame. We were not able to determine the duration of the work disruption or work accommodation that participants reported. In addition, our overall response rate for the survey portion was only 33% and there were statistically significant demographic differences between respondents and non-respondents which suggests our data may not be representative of certain groups such as male cancer survivors. As with all studies that have a qualitative component, interviewer bias may have influenced our findings. We made an effort to reduce this issue by having two trained staff members separately conduct interviews with individual participants.[40] Finally, we were only able to collect limited demographic data for the caregivers who participated in this study. This contributes to our inability to generalize our findings to all caregivers.

### 4.2 Conclusion:

This study documents employment changes among understudied groups, including individuals of lower socioeconomic position. Previous studies have established the need to help cancer survivors navigate issues pertaining to employment.[11, 12, 25] Future studies will need to focus on helping survivors cope financially and mentally with potential changes to their employment and work capabilities in order to prevent long-term negative employment changes as a consequence of cancer. Support for employed, informal caregivers is also needed to prevent long-term financial insecurity for both cancer survivors and caregivers.

## Figures and Tables

**Table 1:** Demographic Characteristics of Employed Cancer Survivors

	Breast-Female (n=197)		Colorectal-Male (n=31)		Colorectal-Female (n=32)		Prostate-Male (n=73)		Total (n=333)	
	No.	%	No.	%	No.	%	No.	%	No.	%
**Sex**										
Male	0	0	31	100	0	0	73	100	104	31.2
Female	197	100	0	0	32	100	0	0	229	68.8
**Ethnicity**										
Non-Hispanic	115	58.4	15	48.4	19	59.4	50	68.5	199	59.8
Hispanic	82	41.6	16	51.6	13	40.6	23	31.5	134	40.2
**Insurance Status**										
Any Medicaid/Uninsured	55	27.9	9	29	10	31.2	6	8.2	80	24
Private Insurance	142	72.1	22	71	22	68.8	67	91.8	253	76
**Education**										
Less than a college degree	98	51	20	64.5	18	58.1	33	45.8	169	51.8
College degree	58	30.2	7	22.6	7	22.6	25	34.7	97	29.8
Graduate or Professional degree	36	18.8	4	12.9	6	19.4	14	19.4	60	18.4
**Annual Household Income**										
Less than 30,000	60	30.5	9	29	14	43.8	7	9.6	90	27
30,000 to 59,000	64	32.5	7	22.6	6	18.8	15	20.5	92	27.6
60,000 to 89,999	33	16.8	8	25.8	6	18.8	11	15.1	58	17.4
More than 90,000	40	20.3	7	22.6	6	18.8	40	54.8	93	27.9
**Debt before cancer diagnosis**										
Less than $1,000	57	29.5	11	36.7	6	19.4	25	34.2	99	30.3
$1,000 – $9,999	66	34.2	7	23.3	17	54.8	16	21.9	106	32.4
$10,000 or More	70	36.3	12	40	8	25.8	32	43.8	122	37.3
**Marital Status**										
Unmarried	75	39.1	9	29	16	51.6	15	20.8	115	35.3
Married	117	60.9	22	71	15	48.4	57	79.2	211	64.7
**Residence**										
Urban	124	63.3	20	64.5	22	68.8	53	72.6	219	66
Rural	72	36.7	11	35.5	10	31.2	20	27.4	113	34
**Comorbidities**										
0	69	35	11	35.5	14	43.8	33	45.2	127	38.1
1	61	31	9	29	8	25	25	34.2	103	30.9
2 or more	67	34	11	35.5	10	31.2	15	20.5	103	30.9
**Surgery**										
No	5	2.5	0	0	4	12.5	30	41.7	39	11.8
Yes	192	97.5	30	100	28	87.5	42	58.3	292	88.2
**Radiation**										
No	108	55.4	21	67.7	24	75	60	83.3	213	64.5
Yes	87	44.6	10	32.3	8	25	12	16.7	117	35.5
**Chemotherapy**										
No	99	50.5	11	35.5	18	56.2	72	100	200	60.4
Yes	97	49.5	20	64.5	14	43.8	0	0	131	39.6

**Table 2:** Summary of Responses to Employment Questions in the CHOICE Survey, *n*=*333*

	Breast-Female (n=197)		Colorectal-Male (n=31)		Colorectal-Female (n=32)		Prostate-Male (n=73)		Total (n=333)	
Question:	No.	%	No.	%	No.	%	No.	%	No.	%
Experienced a work disruption	156	79.2	25	80.6	22	68.8	39	53.4	242	72.7
Change from a full time to part time position	73	37.1	10	32.3	11	34.4	12	16.4	106	31.8
Cancer interfered with physical tasks at work	121	61.4	19	61.3	16	50.0	23	31.5	179	53.8
Cancer interfered with mental tasks at work	115	58.4	12	38.7	17	53.1	11	15.1	155	46.5
Cancer interfered with productivity at work	160	81.2	25	80.6	24	75.0	44	60.3	253	76.0
Was provided a work accommodation	136	69.0	17	54.8	21	65.6	33	45.2	207	62.2
Change from a set schedule to a flexible schedule	84	42.6	14	45.2	13	40.6	10	13.7	121	36.3
Took extended paid time off	116	58.9	12	38.7	18	56.3	29	39.7	175	52.6
Remained at a job to keep health insurance	83	42.1	10	32.3	18	56.3	32	43.8	143	42.9
***Employment among Informal Caregivers***										
Had an informal caregiver	167	84.8	22	71.0	26	81.3	44	60.3	259	77.8
Had an informal caregiver who made a work change	70	35.5	9	29.0	9	28.1	14	19.2	101	30.3
Had an informal caregiver who made a work change for at least 2 months	46	23.4	6	19.4	7	21.9	9	12.3	68	20.4
Had a spouse remain at a job due to concerns about losing health insurance	31	15.7	5	16.1	4	12.5	6	8.2	46	13.8

**Table 3:** Univariable and Multivariable Adjusted Association Between Sociodemographic Characteristics and Cancer Survivors who Experienced a Work Disruption or Work Accommodation

	Work Disruption (n=242)						Work Accommodation (n=207)					
	Unadjusted			Adjusted			Unadjusted			Adjusted		
Characteristic	OR	95% CI	p-value	OR	95% CI	p-value	OR	95% CI	p-value	OR	95% CI	p-value
												
**Age at diagnosis**	0.41	0.17,1.01	0.05	0.97	0.91,1.03	0.300	0.21	0.68,0.67	0.01	0.90	0.84,0.96	0.002
**Cancer site**												
Breast	1.00	ref		1.00	ref		1.00	ref				
Colorectal	0.85	0.32,2.29	0.75	0.86	0.30,2.43	0.780	0.96	0.38,2.39	0.93			
Prostate	0.39	0.17,0.89	0.03	0.38	0.15,0.98	0.050	0.73	0.30,1.77	0.48			
**Number of comorbid conditions**												
0	1.00	ref					1.00	ref		1.00	ref	
1	1.39	0.60,3.26	0.44				1.28	0.55,2.95	0.57	2.48	0.92,6.66	0.070
≥2	1.95	0.79,4.81	0.15				1.28	0.56,2.89	0.56	3.59	1.27,10.1	0.020
**Education**												
≤High School Diploma or Some College	1.00	ref					1.00	ref				
College Degree	1.05	0.46,2.39	0.91				1.6	0.72,3.55	0.25			
>College or Professional Degree	1.08	0.40,2.90	0.88				5.00	1.13,22.1	0.03			
**Insurance**												
Any Medicaid/Uninsured	1.00	ref		1.00	ref		1.00	ref				
Private Insurance	0.56	0.22,1.41	0.22	0.33	0.09,1.17	0.090	2.63	1.29,5.36	0.01			
**Income**												
Less than $30,000	1.00	ref		1	ref		1.00	ref		1.00	ref	
$30,000 – $69,999	1.68	0.63,4.48	0.30	3.06	0.91,10.25	0.070	3.82	1.57,9.34	0.003	5.29	1.87,14.97	0.002
$70,000 or More	0.79	0.34,1.86	0.59	1.77	0.51,6.08	0.370	3.06	1.32,7.07	0.01	6.07	2.14,17.20	0.001
**Debt**												
Less than $1,000	1.00	ref					1.00	ref		1.00	ref	
$1,000 – $9,999	1.99	0.74,5.33	0.17				1.29	0.47,3.56	0.62	1.08	0.36,3.23	0.890
$10,000 or More	1.12	0.49,2.57	0.79				0.64	0.27,1.53	0.32	0.38	0.14,1.04	0.060
**Ethnicity**														
Non-Hispanic	1.00	ref		1.00	ref		1.00	ref				
Hispanic	0.52	0.25,1.07	0.08	0.45	0.20,1.00	0.050	0.80	0.40,1.59	0.52			
**Marital status/sex**												
Married male	1.00	ref					1.00	ref				
Non-married male	1.13	0.28,4.65	0.86				0.51	0.12,2.05	0.34			
Married female	1.79	0.72,4.44	0.21				1.51	0.54,4.18	0.43			
Non-married female	1.97	0.72,5.34	0.18				0.74	0.28,1.95	0.54			
**Geographic location**												
Urban	1.00	ref					1.00	ref				
Rural	0.82	0.39,1.72	0.60				0.94	0.46,1.92	0.86			

**Table 4:** Demographic Characteristics of Survivors and Informal Caregivers, *n*=*28*

	Survivor (n=17)	
	Median	(min, max)
**Current age**	57.5	(40, 64)
	n	%
**Gender**		
Male	2	11.8
Female	15	88.2
**Ethnicity**		
Non-Hispanic	12	70.6
Hispanic	5	29.4
**Cancer site**		
Breast	12	70.6
Prostate	1	5.9
Colorectal	4	23.5
**Insurance Status**		
Private Insurance	10	58.8
Any Medicaid	5	29.4
Uninsured	2	11.8
**Annual Household Income**		
Less than $30,000	6	35.3
$30,000 – $49,999	4	23.5
$50,000 or More	7	41.2
	Informal Caregiver (n=11)	
	Median	(min, max)
**Time since Diagnosis of Care Recipient**	*6*	*(3,9)*
	n	%
**Gender**		
Male	6	54.5
Female	5	45.5
**Relationship to Survivor**		
Spouse/Partner	10	90.9
Sibling	1	9.1
**Cancer Site of Care Recipient**		
Breast	7	63.6
Prostate	2	18.2
Colorectal	2	18.2

**Table 5:** Summary of Qualitative Themes and Examples

Theme	Example	Type of Participant
Employment Disruption	Unable to perform mental or physical tasks	Survivors
Employment Accommodation	Flexible schedules	Surviviors & Caregivers
Remaining at a Job to Keep Insurance	Disability & Health Policies	Survivors
Coping Behaviors and Strategies	SNAP &Go-fund-me	Surviviors & Caregivers
